# Frequency and characteristics of errors by artificial intelligence (AI) in reading screening mammography: a systematic review

**DOI:** 10.1007/s10549-024-07353-3

**Published:** 2024-06-09

**Authors:** Aileen Zeng, Nehmat Houssami, Naomi Noguchi, Brooke Nickel, M. Luke Marinovich

**Affiliations:** 1 The Daffodil Centre, The University of Sydney, a Joint Venture with Cancer Council New South Wales, Sydney, NSW Australia; 2 School of Public Health, Faculty of Medicine and Health, The University of Sydney, Sydney, NSW Australia; 3 Wiser Healthcare, Sydney School of Public Health, Faculty of Medicine and Health, The University of Sydney, Sydney, NSW Australia; 4 Sydney Health Literacy Lab, Sydney School of Public Health, Faculty of Medicine and Health, University of Sydney, Sydney, NSW Australia; 5 Westmead Applied Research Centre and Faculty of Medicine and Health, The University of Sydney, Westmead, NSW Australia

**Keywords:** Artificial intelligence, Breast cancer, Diagnostic errors, Population screening

## Abstract

**Purpose:**

Artificial intelligence (AI) for reading breast screening mammograms could potentially replace (some) human-reading and improve screening effectiveness. This systematic review aims to identify and quantify the types of AI errors to better understand the consequences of implementing this technology.

**Methods:**

Electronic databases were searched for external validation studies of the accuracy of AI algorithms in real-world screening mammograms. Descriptive synthesis was performed on error types and frequency. False negative proportions (FNP) and false positive proportions (FPP) were pooled within AI positivity thresholds using random-effects meta-analysis.

**Results:**

Seven retrospective studies (447,676 examinations; published 2019–2022) met inclusion criteria. Five studies reported AI error as false negatives or false positives. Pooled FPP decreased incrementally with increasing positivity threshold (71.83% [95% CI 69.67, 73.90] at Transpara 3 to 10.77% [95% CI 8.34, 13.79] at Transpara 9). Pooled FNP increased incrementally from 0.02% [95% CI 0.01, 0.03] (Transpara 3) to 0.12% [95% CI 0.06, 0.26] (Transpara 9), consistent with a trade-off with FPP. Heterogeneity within thresholds reflected algorithm version and completeness of the reference standard. Other forms of AI error were reported rarely (location error and technical error in one study each).

**Conclusion:**

AI errors are largely interpreted in the framework of test accuracy. FP and FN errors show expected variability not only by positivity threshold, but also by algorithm version and study quality. Reporting of other forms of AI errors is sparse, despite their potential implications for adoption of the technology. Considering broader types of AI error would add nuance to reporting that can inform inferences about AI’s utility.

**Supplementary Information:**

The online version contains supplementary material available at 10.1007/s10549-024-07353-3.

## Introduction

Artificial intelligence (AI), a rapidly evolving field of data science in which computer algorithms are developed to perform complex tasks, has been applied to screening mammography for the early detection of breast cancer with the aim of improving outcomes for screening participants [1]. AI has the potential to identify cancers in mammograms that are not perceptible to human readers, thereby potentially increasing the sensitivity of screening and improving outcomes for women through initiation of treatment for early-stage disease. Other proposed benefits of AI include fewer false positive findings that lead to anxiety and unnecessary investigations, and workforce efficiencies for screening programmes that may translate to lower programme costs and improvements in the screening experience for women. Such benefits assume that AI performs at least as accurately as human readers in detecting breast cancer, and research has therefore focussed on evaluating the comparative accuracy of algorithms and human readers. However, there is recognition that even when algorithms exhibit high performance in selected research datasets, AI errors in cancer detection (false positives, FP; false negatives, FN) may be greater when algorithms are applied in “real-world” settings or transferred between populations [2]. Furthermore, technological updates can produce subtle changes to medical images which may not be obvious to humans but can alter the AI’s output [3]. Such errors may be difficult to detect and explain by humans [4] and may strongly influence decision making by human readers (automation bias) [5]. Given the theoretical ease for AI algorithms to be scaled up and applied to large populations, unpredictable or unexpected errors may lead to harmful consequences.

Beyond the potential for FP or FN cancer findings, the concept of AI “error” in automated mammography interpretation has not been clearly delineated. Other types of error may include a (true positive) cancer detected in the wrong location, or technical errors that result in the algorithm failing to process images or generate a result. Earlier systematic reviews presented AI error as FP and FN, which is consistent with the focus on test accuracy in the literature [6–8]. However, imaging or lesion features associated with these FP and FN were not elaborated, and other potential forms of error were not reported. At present, it is unclear what forms of AI error are reported in the literature, as well as the frequency and lesion or imaging features of these AI errors.

In this study, we aim to identify the range of outcomes that have been reported as AI errors; quantify the frequency AI errors; and to describe the study, imaging, or lesion features associated with AI errors in practice. To meet this objective, we performed a systematic literature review of external validation studies of AI algorithms for independent mammographic interpretation using real-world screening data.

## Materials and methods

This systematic review was performed and reported in accordance with Preferred Reporting Items for Systematic Reviews and Meta-Analyses of Diagnostic Test Accuracy (PRISMA-DTA) statement [9]. The review protocol was registered in the International Prospective Register of Systematic Reviews (PROPSERO) (CRD42022340331).

### Information sources and literature search

A literature search was conducted without language restrictions for diagnostic accuracy studies published from 1 January 2010 to 11 July 2022. To capture contemporary AI algorithms, the search was limited from January 2010, coinciding with technical and hardware developments that facilitated efficient processing of machine learning [10]. Databases searched include MEDLINE, EMBASE, SCOPUS and a pre-print database, ArXiv. We reviewed reference lists of relevant systematic reviews to identify the additional studies. Details of the search strategy are listed in Online Resource 1.

### Study selection

One reviewer (A.Z.) independently screened titles and abstracts and subsequent full-text articles against eligibility criteria (Online Resource 2). A second reviewer (M.L.M.) independently screened a 25% sample of titles and abstracts and the final set of included full-text studies for quality assurance.

Eligible study designs were external validation studies performed in population breast cancer screening settings where the AI algorithm acted as an independent reader (defined as a standalone system for replacement of radiologist reading, or as a pre-screen to triage whether the mammogram requires further review by a radiologist). Where studies included both conventional mammography and tomosynthesis, data on mammograms only were included.

Studies were excluded if more than 5% of included mammograms were incomplete; AI was used as a prediction tool (e.g. cancer subtypes, lesion characteristics or risk) or to assist radiologist reading (meaning the read was not solely from the AI algorithm); or AI was implemented for other imaging types (e.g. magnetic resonance imaging or ultrasound). Studies were excluded if outcomes did not include AI errors.

### Data extraction and risk of bias assessment

Two reviewers (A.Z. and N.N.) independently extracted data on a pre-designed standardised collection form. Data that were systematically extracted included study design and setting, population characteristics, commercial availability, frequency and characteristics of pre-specified AI errors [false positives (FP), false negatives (FN), location error, technical error] and reference standard. Other forms of AI error were extracted when reported. FP was defined as incorrect presence of a suspicious finding (in cases where no cancer is found). FN was defined as cancer not detected by AI but detected by radiologist(s) or found at follow-up. Location error was defined as correct diagnosis of cancer, but the region of interest indicated by AI does not correspond with cancer location. Technical error was defined as failure of AI to process and interpret the mammogram or output a finding.

From studies reporting AI accuracy, we extracted raw values to derive 2 × 2 tables cross-classifying the AI result (positive or negative) and the reference standard finding (cancer present or absent). From these values, we calculated false positive proportions (FPP) and false negative proportions (FNP) per study (FP or FN divided by total number of examinations). When studies reported data at comparable positivity thresholds (including multiple possible thresholds per study), we extracted data and calculated FNP and FPP, and sensitivity and specificity estimates at those thresholds. Only common positivity thresholds across studies were reported.

Two reviewers (A.Z. and M.L.M.) independently assessed methodological quality using the Quality Assessment of Diagnostic Accuracy Studies (QUADAS-2) tool modified to the review question and QUADAS-AI preliminary extension [11] (Online Resource 3). Risk of bias of individual studies was assessed under four domains including (i) patient selection, (ii) index test, (iii) reference standard and (iv) flow and timing. The first three domains were assessed in terms of concerns regarding applicability. Reference standards were recorded to assess comparability across studies. All discrepancies were resolved by discussion and consensus.

### Data synthesis

Narrative synthesis was conducted because of methodological variations between studies. FPP, FNP, location, technical and other errors were tabulated. FNP and FPP estimates and their 95% Wald confidence intervals (CIs) were plotted in a forest plot. Estimates were pooled within each positivity threshold using inverse variance random-effects meta-analysis with the restricted maximum likelihood estimator [12, 13]. Tests for subgroup differences between thresholds were not calculated because data in each subgroup were not independent.

Sensitivity and specificity of single and consensus readers were plotted against AI positivity thresholds (when reported) in receiver operating characteristic (ROC) space to complement FPP and FNP estimates.

Analyses were undertaken using the *metafor* package and visual summaries were generated using the *ggplot2* package [14, 15] in R version 4.2.2 (R Project for Statistical Computing in Vienna, Austria).

## Results

**Fig. 1:**
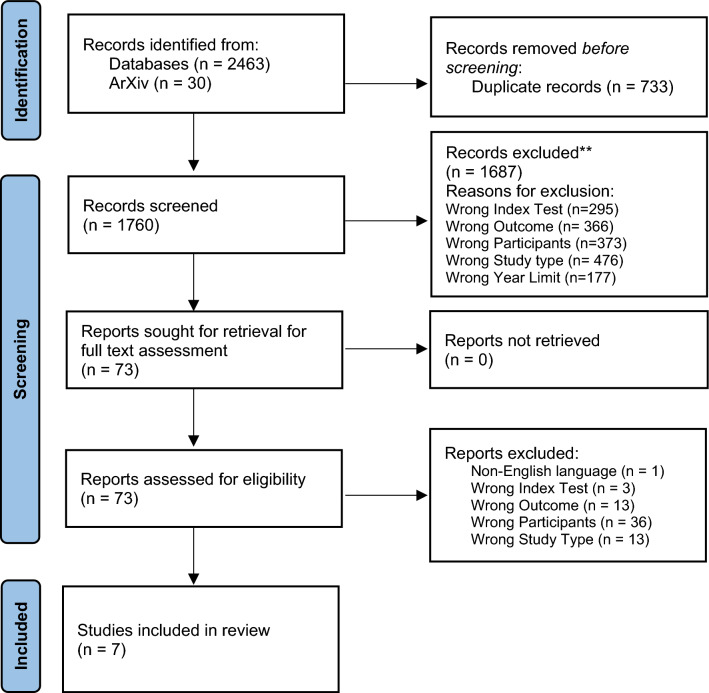
Preferred Reporting Items for Systematic Review and Meta-Analyses flowchart (PRISMA) flowchart

After deduplication, 1760 unique results were screened, of which 73 potentially eligible full-text articles were assessed. Seven studies were included in this review [16–22]. Figure 1 summarises the screening and eligibility process and documents reasons for exclusion.

### Study characteristics of included studies

Characteristics of the seven included studies, comprising 447,676 examinations, are presented in Table 1. One study reported AI error as the location of false markings on the mammogram [20]. The remaining six studies reported AI sensitivity and specificity [16–19, 21, 22], and five of those reported AI errors as FNR and FPR according to the positivity threshold applied [16–19, 22]. One study reported additional error information as AI technical failures [19].

**Table 1:** Summary of study characteristics of included studies

Study	Study design	Modality of AI	Model; version	Positivity threshold	Population	Comparator	Reference standard
Balta (2020) [16]	Retrospective cohort study with consecutive screens (accuracy of classifying into low and high-risk categories) Time of enrolment: January–November 2018	AI as triage	Transpara; V1.6.0, screen point medical	Retrospective multiple (*n* = 10) threshold Main threshold: 7 Low risk: 1–7 then single read High risk: 8–10 then consensus read^a^ (10 = highest suspicion of cancer) Threshold Scale: 1–10	17,895 examinations (114 screen-detected cancers) Prevalence of cancer: 0.64% Age: NR Country: Germany	Radiologist; single and consensus read	Cancer presence: histopathology of cancer
Lauritzen (2022) [19]	Retrospective Cohort study with consecutive screens (accuracy of classifying into low and high-risk categories) Time of enrolment: January 2014–December 2015	AI as triage	Transpara; V1.7.0, Screen Point Medical	Prospective multiple (*n* = 3) threshold Main threshold: 5 0 ≤ Score < 5 then no human-reading Moderate Risk: 5 ≤ Score ≤ 9.989 then consensus read Suspicious: 9.989 < Score ≤ 10 then recall (10 = highest suspicion of cancer) Threshold Scale: 0–10	114 421 examinations from 114 421 women (791 screen-detected cancers and 327 interval cancers) Prevalence of Cancer: 0.98% Mean Age: 59 years ± 6 (SD) (50–69 years) Country: Denmark	Radiologist (average 7.1 years of high volume reads); consensus read	Cancer Presence: Danish Cancer Registry (linked with Histopathology of Cancer); Follow up period and screening interval: 2 years
Lang (2021) [17]	Retrospective Cohort Study with consecutive screens (accuracy of classifying into low and high-risk categories) Time of enrolment: February 2012–May 2015	AI as triage	Transpara; V1.4.0, Screen Point Medical	Prospective Single threshold Main threshold: 5 Score ≤ 2 then no human-reading Low risk: 1–5 High risk: 6–10 then reading by radiologists (10 = highest suspicion of cancer) Threshold Scale: 1–10	9581 examinations from 9581 women (68 screen-detected cancers) Prevalence of cancer: 0.71% Mean age: 57.6 years ± 9.5 (SD) (40–74 years) Country: Sweden	No comparator	Cancer presence: surgical or core histopathology of Cancer; Regional cancer registry
Raya-Povedano (2021) [22]	Retrospective Cohort Study with consecutive screens (accuracy of classifying into low and high-risk categories) Time of enrolment: January 2015–December 2016	AI as triage	Transpara; V 1.6.0, Screen Point medical	Prospective Single Threshold Main threshold: 7 Low risk: 1–7 then no human-reading High risk: 8–10 then consensus read (10 = highest suspicion of cancer) Threshold Scale: 1–10	15,987 examinations from 15,986 women (98 screen-detected cancers and 15 interval cancers) Prevalence of cancer: 0.71% Mean Age: 58 years ± 6 (SD); (50–69 years) Country: Spain	Radiologist (3–15 years’ experience); consensus read (as per original screening from Cordoba Tomosynthesis Screening Trial)	Cancer presence: histopathology of cancer and interval cancer diagnosis; screening interval & follow period: 2 years
Larsen (2022) [18]	Retrospective Cohort Study with consecutive screens (accuracy of a read) Time of enrolment: October 2009–December 2018	Standalone AI System	Transpara 1.7.0, Screen Point Medical	Prospective single threshold Main threshold: 9 & Retrospective Multiple Threshold (matched reader sensitivity and specificity (Raw Score ≥ 9.13 OR ≥ 9.43)) (10 = highest suspicion of cancer) Threshold Scale: 1–10	122,969 examinations from 47,877 women (752 screen-detected cancers and 205 interval cancers) Prevalence of cancer: 0.78% Mean age: 60 years ± 6 (SD) Country: Norway	Radiologist (1–20 + years’ experience); consensus read	Cancer Presence: Cancer Registry AND Screening Interval: 2 years; Follow Up period from 1st screen: 6–24 months (after recall) or within 2 years after a negative screen
Mayo (2019) [20]	Retrospective Cohort Study with consecutive screens (accuracy of detecting False Positive Markings) Time of enrolment: January 2013–March 2013	Standalone AI System	cmAssist (protype AI-CAD, CureMerix, La Jolla, CA)	NR	245 examinations (3 cancers within follow-up period) Prevalence of cancer: 1.22% Age: 40–90 years Country: USA	Computer-Aided Detection (CAD)	Cancer Location: Clinic Review of established location of the biopsied lesion (screen negatives confirmed by follow period ≥ 2 years)
Schaffter (2020) [21]	Retrospective Cohort Study with consecutive screens (accuracy of a read) Time of enrolment: April 2008–December 2012	Standalone AI System	Ensemble model	Retrospective single threshold specificity threshold: 77.1% (single reader); 83.9% (consensus reader)	166,578 examinations from 68,008 women (780 cancers within 1 year follow-up) Prevalence of cancer: 1.1% Mean Age: 53.3 year ± 9.4 (SD) Country: Sweden	Radiologist, single and consensus read	Cancer Presence: Histopathology of Cancer; Screening Interval: 2 years Follow Period from first screen: 1 year

*AI* artificial intelligence; *CAD* computer-aided diagnosis; *NR* not reported; *SD* standard deviation

Two studies evaluated datasets on real-world screening populations from Sweden, and one each evaluated screening populations from Denmark, Norway, Germany, Spain, and the United States (US). Evaluation datasets were sourced from screening programmes [18, 19, 21], sub-cohorts of randomised controlled trials [17, 22] and specialist cancer centres [16, 20]. Screening mammograms all had 4 views (2 views per breast). Mammogram units were Siemens Mammomat [17–19] or Hologic Selenia [20, 22], and one study reported the use of both [16]. All were retrospective cohort studies with consecutive screens. Years of enrolment ranged from 2008 to 2018. Study-level mean age of the women ranged between 53 and 60 years.

For six studies reporting on FPR and FNR and/or sensitivity and specificity, two studies used a reference standard of screen-detected cancers only [16, 17], and four included interval cancers (in addition to screen-detected cancers) with follow-up of either 12 months [21] or 24 months [18, 19, 22]. Cancer prevalence in studies with screen-detected cancers only ranged between 0.64 and 0.71%, whereas this was from 0.71 to 1.22% for studies that included screen-detected and interval cancers. The reference standard in an additional study reporting cancer location was clinical review of established biopsied cancer location (all screen-detected; ≥ 2 year follow-up confirmed no interval cancers). An AI cancer marking was considered correct if its location intersected with the geometric centre of ground-truth (radiologists’) region of interest (ROI) [20].

Four studies using AI for triage reported “high”- or “moderate”-risk cases would be reviewed by radiologists [16, 17, 19, 22]. “Low-risk” cases (i.e. case deemed as low suspicion of cancer) would have no human-reading or reading by a single radiologist. AI performance was compared to radiologists (single or consensus reading) with 3 to 15 years of experience [16, 19, 22]. Three studies used standalone AI to evaluate its accuracy compared to either double reading (1–20 + years’ experience) [18, 21] or computer-aided detection (CAD) to reduce false positive markings on mammograms [20].

Table 2 summarises the risk of bias and applicability concerns of included studies. Overall, four studies had high risk of bias or applicability concerns in at least one of the four domains [16, 17, 20, 21]. Two studies had high risk of bias and applicability concerns for the reference standard [16, 17] and three studies had unclear risk of bias for patient selection [17, 20, 22]. Four studies had either high or unclear risk of bias in flow and timing [16, 17, 20, 21].

**Table 2:** “Traffic Light Plot” of overview of risk of bias and applicability of included studies

Study reference	Risk of bias				Applicability concerns		
	Patient selection	Index test	Reference standard	Flow and timing	Patient selection	Index test	Reference standard
AI for triage (4 studies)							
Balta (2020) [16]							
Lang (2021) [17]							
Lauritzen (2022) [19]							
Raya-Povedano (2021) [22]							
Standalone AI systems (3 studies)							
Larsen (2022) [18]							
Mayo (2019) [20]							
Schaffter (2020) [21]							

High risk

Unclear risk

Low risk

### Characteristics of AI tools and positivity thresholds

Of the six studies reporting FP and FN errors, five evaluated different versions (V1.4.0 to V1.7.0) of a commercially available algorithm (Transpara, Screen Point Medical) [16–19, 22] and one used an ensemble model [21] (Online Resource 4). An additional study reporting location errors used a prototype AI-CAD system [20].

In studies applying AI for triage, two used a single prospective threshold [17, 22], one used multiple prospective thresholds [19] and one study used multiple retrospective thresholds [16]. The most commonly reported thresholds were Transpara Score 5 [17, 19] or 7 [16, 22], where 10 equates to the highest suspicion of cancer on a scale of 0–10.

In studies using standalone AI, one used a single prospective threshold (Transpara Score 9) in addition to retrospective thresholds [18] and one used a retrospective single threshold to match radiologists’ specificity [21]. The study that assessed the location of any AI markings on the mammogram did not specify an AI positivity threshold [20].

### Reported AI errors and associated factors

Table 3 presents the frequency and features associated with reported AI error.

**Table 3:** Features associated with reported AI errors

Study	Reported error (N)	Imaging or lesion or other features associated with error
Balta (2020) [16]	Threshold: Transpara Score = 7 False Positives (6135/ 17,896); False Negatives (9/17896)	NR
Lang (2021) [17]	Threshold: Transpara Score = 5 score: False Positives (4438/9581); False Negatives (7/9581)	At Threshold: Transpara Score = 5 score: Overall, less cancers (7/9581) were *missed* by AI than detected by AI (61/9581) All *7 cancers* missed by AI were invasive cancers 3 of 7 *missed* cancers were small ($\le$ 7 mm), low grade invasive tubular carcinomas 2 of 7 *missed* cancers were large (20 mm) invasive ductal cancers and 1 of 7 *missed* cancers was an invasive ductal cancer with unknown size 1 of the 7 *missed* cancers was invasive lobular cancer (20 mm) 6 of the 7 AI *missed* cancers had a radiographic appearance of spiculated mass and were from women with dense breasts (BIRADS Density C and D) Compared to the AI detected cancers (61/9581): majority were invasive ductal cancer (30/9581) followed by DCIS (11/9581), invasive lobular cancer (10/9581), invasive tubular Cancer (7/9581) and Other (e.g. papillary carcinoma, apocrine tumour) (3/9581)
Lauritzen 2022 [19]	Threshold: Transpara Score = 5: False Positives (41,909/114421); False Negatives (105/114421)	No reported technical failure: AI system was able to process all the available mammograms
Raya-Povedano 2021 [22]	Threshold: Transpara Score = 7: False Positives (4450/ 15,987); False Negatives (13/15987)	At Threshold: Transpara Score = 7 score: All AI *missed* cancers were interval cancers (13 of 13)
Larsen 2022 [18]	Threshold: Transpara Score = 9 score False Positives (11,638 /122969); False Negatives (212 /122969)	At Threshold: Transpara Score = 9 score: Overall, less cancers (212/122969) were *missed* by AI then detected by AI (745/122969) Majority of AI *missed* cancers were interval cancers (113/212) compared to screen-detected cancers (99/212) Compared to AI detected cancers, majority were *screen-detected* cancers (653/745) than interval cancers (92/745) Larger proportion of the *missed* AI cancers were invasive cancer (187/212) compared to DCIS (25/212) Similar to AI detected cancers (745/122969): Majority of AI detected cancers were invasive (623/745) than DCIS (122/745) Median tumour diameter of all AI *missed* cancers (7-25 mm) reported to be smaller than all AI detected cancers (9-30 mm)
Mayo 2019 [20]	Location Error- False Positive Marks (126/ 245)	Radiographic Features: Calcification accounted for 0.07 (95% CI 0.041, 0.11) False Positive Mark per Image (FPPI) Masses accounted for 0.22 (95% CI 0.18, 0.26) FPPI Reduction of false positive markings consistent across all BIRADS density categories from fatty to extremely dense False Positive Marks (8 of the 18 false positive recalls) were ultimately confirmed as benign lesions following follow-up of 2 years

Abbreviations: AI = artificial intelligence; BIRADS = Breast Imaging Reporting and Data System; DCIS = ductal carcinoma in situ; FPPI = false positive mark per image; NR = not reported

Six studies reported sensitivity and specificity [16–19, 21, 22], five of which also reported AI error as false negatives and false positives according to comparable positivity thresholds [16–19, 22]. One study reported on AI error as technical failure which was defined as failure of the AI to process mammograms[19]. One study referred to location error as an AI cancer marking that is incorrectly highlighted on a mammogram (i.e. false positive marks) [20].

#### False positive proportion (FPP) and false negative proportion (FNP)

**Fig. 2:**
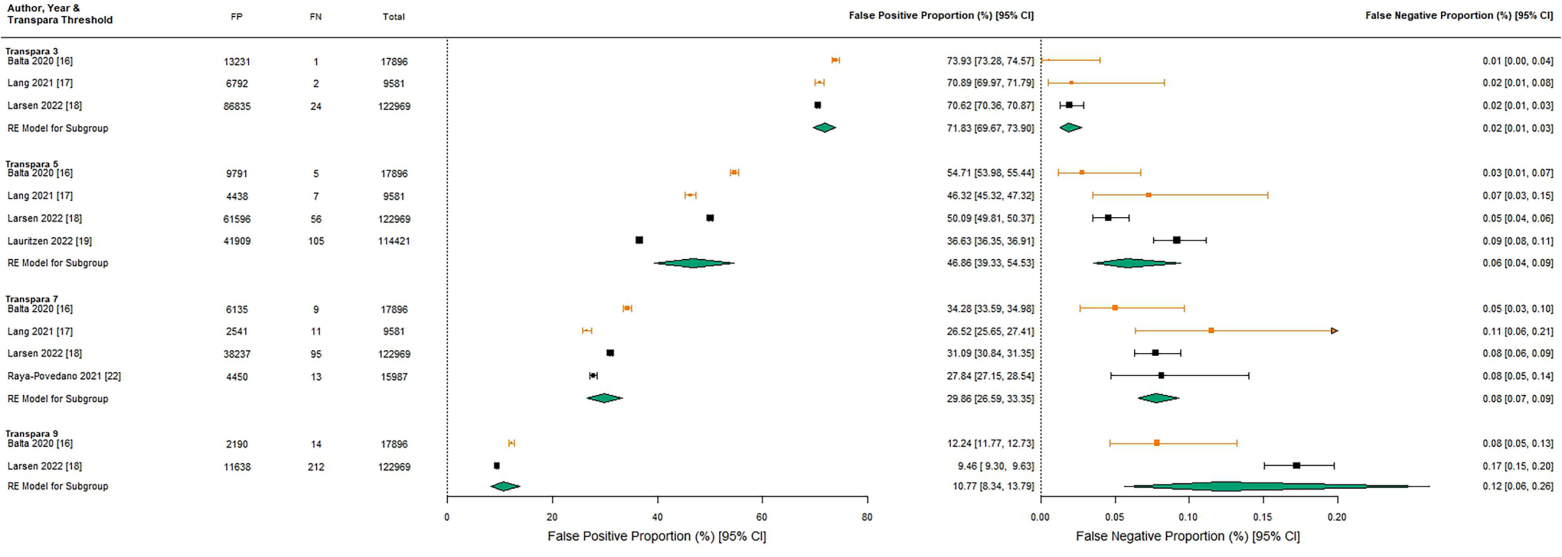
Forest plots of false positive proportion and false negative proportion by Transpara threshold. Estimates for studies that only include screen-detected cancer are denoted in orange

Pooled FPP decreased incrementally with increasing Transpara threshold (71.83% [95% CI 69.67, 73.90] at Transpara 3 to 10.77% [95% CI 8.34, 13.79] at Transpara 9). The most commonly reported prospective triage thresholds were Transpara 5 (pooled FPP 46.86% [95% CI 39.33, 54.53]) and Transpara 7 (pooled FPP 29.86% [95% CI 26.59, 33.35) (Fig. 2).

The pooled FNP increased incrementally from 0.02% [95% CI 0.01, 0.03] at Transpara 3 to 0.12% [95% CI 0.06, 0.26] (Transpara 9), consistent with a trade-off with FPP.

There was heterogeneity within Transpara thresholds, reflecting study-level differences in Transpara version and reference standard (Fig. 2, Online Resource 5). For studies using later versions of Transpara (V1.6.0 and V1.7.0), FPP was lower (and FNP higher) within each threshold when the reference standard included screen-detected and interval cancers [16], compared with studies including only screen-detected cancers in the reference standard [18, 19, 22]. One study that evaluated an earlier Transpara version (V1.4.0) included only screen-detected cancers in the reference standard [17]; it reported lower FPP and higher FNP relative to a study using the same reference standard and a later Transpara version [16].

Table 3 describes the lesion or imaging features associated with AI missed cancers (i.e. FN). Three studies reported on the lesion or imaging characteristics associated with FN, each at different Transpara scores (i.e. 5, 7, 9). Two studies reported that the majority of FN were invasive cancers (88–100%) [17, 18]. A majority of FN (53–100%) were interval cancers [18, 22]. One study reported FN cancers generally had a radiographic appearance of spiculated mass and were in Breast Imaging Reporting and Data System Density C and D breasts [17]. Two studies reported that median tumour size for cancers missed by AI ranged from ≤ 7 to 25 mm [17, 18]. When compared to AI detected cancers, the majority of these (77–84%) were also invasive cancers [17, 18].

#### Sensitivity and specificity

**Fig. 3:**
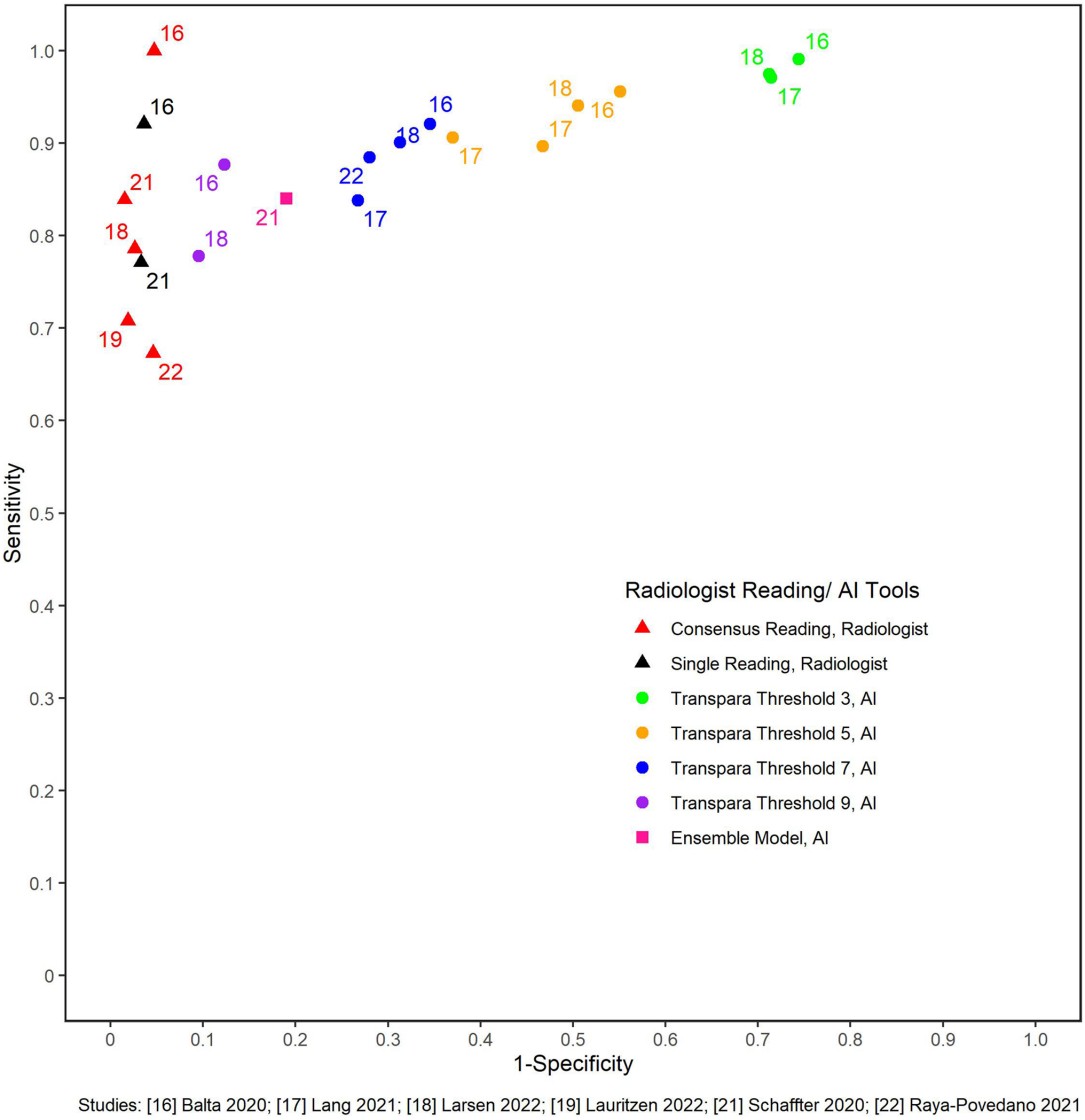
Study estimates of sensitivity and specificity in receiver operating characteristics space for AI tools vs single or consensus reading by radiologists

In studies that compared AI performance to radiologists, two reported on single reading [16, 21] and four [16, 18, 21, 22] reported on consensus reading. Transpara was the AI tool used in five of these studies (the other used an ensemble system [21]). As expected, we observed a trade-off with higher specificity and lower sensitivity as the Transpara positivity threshold increased. Regardless of single or consensus reading, the radiologists’ specificity remained consistent relative to the varied specificity and sensitivity at different AI positivity thresholds (Fig. 3). The range in sensitivity of a single reader is comparable to Transpara Score 7 (0.83–0.92) or 9 (0.77–0.88).

#### Other types of error

Two studies reported other forms of AI errors. One study reported that no technical failures were encountered in which the AI model failed to process mammograms [19]. A second study investigated the location of AI false marking on the mammogram [20]. Location error was weakly associated with radiographic features including calcification or masses. Eight of the 18 location errors were ultimately confirmed as benign lesions. No other forms of AI error were identified from included studies.

## Discussion

This systematic review of externally validated AI algorithms for cancer detection in breast screening identified relatively few studies that report AI errors. Four types of AI errors were identified, the most commonly reported being false positive and false negative findings, which is consistent with a focus on diagnostic accuracy in studies of AI in breast cancer screening [16–19, 22]. Previous systematic reviews have assessed the diagnostic accuracy of AI in external validation studies; however, none have reported on AI error in detail [6–8]. This review is a novel attempt to report findings and imaging features associated with AI errors and identify other types of error. Technical and location errors were reported relatively infrequently and inconsistently, despite their importance in establishing the utility of AI in population breast cancer screening practice.

The findings highlight factors relating to algorithm, study, and imaging characteristics that may plausibly influence the FPP and FNP of AI in the breast screening context. The exploration of multiple AI positivity thresholds showed the expected trade-off between FPP and FNP, with progressively lower FPP (and higher FNP) as the positivity threshold increased. However, there was considerable heterogeneity of FPP and FNP within thresholds. Between-studies comparisons suggested that the frequency of these errors varied according to the version of the AI algorithm. Lower FNP was observed with more recent (v1.6.0 and 1.7.0) compared with earlier (v1.4.0) versions of Transpara, suggesting that improvements in AI over time have resulted in a lower likelihood of errors leading to missed cancers. However, a corresponding increase in FPR was also found, indicating that technical improvements to enhance AI sensitivity have the potential to result in increased recall. Studies that have integrated AI into the screen-reading workflow as an adjunct to radiologist reading have used recent Transpara versions[18, 23]; absent comparison with earlier versions, it is unclear if these observed differences in AI error rates may have translated to increased cancer detection and recall over time.

The comparisons also highlight the importance of appropriately defining the reference standard to classify FP and FN results. Studies that included both screen-detected and interval cancers reported lower FPP for AI compared with studies including screen-detected cancers only, logically reflecting the limitation of the latter design in validating true positive AI results that are deemed negative by radiologists [6]. “Missed” interval cancers by AI also contributed to higher FNP in such studies. Incompleteness of interval cancer ascertainment has been identified as source of potential bias in studies of AI [6, 8], with empirical studies showing an inflation of overall accuracy [2]. Studies investigating AI errors should, at minimum, include all interval cancers (ideally through registry linkage to minimise the potential for bias) [8]; extended follow-up should also be considered [8, 24], acknowledging the desirability of aligning follow-up with screening intervals which may differ between settings.

The above suggestion that studies of AI for mammography screening should use sufficient follow-up to include interval cancers is reinforced in the study from Larsen et al. [18] which used cancer registry linkage to ascertain interval cancers—it showed more of the cancers missed by the AI were interval (than screen-detected) cancers. It also showed that the AI was more likely to miss a smaller tumour than a larger tumour on the mammogram, evidenced in a median tumour diameter of cancers *missed* by AI ranging between 7 and 25 mm, whereas those correctly detected by the AI ranged between 9 and 30 mm.

Technical AI errors—in which the algorithm fails to generate output—may have important implications for implementation of AI in screening programmes. Such failures require remediation in the workflow, and systematic failures have the potential to have disproportionate impacts on different sub-populations [3]. Location errors—where AI identifies abnormality in an incorrect location of the breast—have potentially adverse clinical consequences for women and may erode radiologists’ confidence in AI findings. However, it should be noted that at present, there is no gold standard for defining these ‘location-specific’ errors which require clinical (imaging) review and subjective judgement, and this is an area worthy of further exploration. The one study identified in this review referred to location error as an AI cancer marking that is incorrectly highlighted on a mammogram and used a retrospective clinical review process [20]. Breast imaging fellowship-trained radiologists generated a region of interest to establish the location of the biopsied lesion, and AI markings were considered to be correct if the geometric centre lay within the region of interest.

Despite the importance of understanding the frequency and nature of both technical and location errors that occur when AI reads mammography, this review found that they were reported infrequently. In the case of technical errors, these were reported in only one instance to confirm the absence of such errors [21]. Additional emphasis on enumerating and describing such AI errors would be a valuable complement to the current research focus on accuracy (including FP and FN errors), enabling better understanding of the potential impact of AI on screening workflow and clinical outcomes. Improving knowledge on these issues is highly relevant for potential implementation of AI in breast screening practice, noting that women have high expectations that AI will improve mammography screening accuracy and outcomes [25].

This review did have limitations. Firstly, it focused on AI as standalone reader, not as an aid to reader interpretation. This may have limited the search strategy to exclude studies that are more likely to report location errors. However, these errors have been reported mostly in reader studies using cancer-enriched datasets and may not be generalisable to population breast cancer screening settings [7]. Furthermore, the review interprets between-study comparisons to explain heterogeneity in error estimates. Although the observed differences in FPP and FNP are in the expected directions, there are likely to be clinical and methodological differences between studies beyond those considered in our analyses. Within-study comparisons would provide stronger evidence from which to draw inferences. Where possible, authors should be encouraged to facilitate such comparisons by reporting FP and FN errors for screen-detected and interval cancers separately, and multiple follow-up times for ascertaining interval cancers [2].

## Conclusions

Current evidence on AI algorithms in breast cancer screening demonstrates that false positives and false negatives are the predominantly reported forms of AI errors, which is consistent with the focus on diagnostic accuracy in the literature. Further reporting of other types of errors, including technical errors, could provide a better understanding of AI’s utility in breast screening practice. Further studies on AI errors using real-world data could also allow future systematic reviews to explore plausible factors (e.g. clinical or radiological characteristics) associated with errors that are generalisable to real populations. This could complement co-existing AI accuracy research, to ensure the safe integration of AI into future screening practice.

### Supplementary Information

Below is the link to the electronic supplementary material.

Supplementary file1 (DOCX 39 KB)

## Data Availability

Data sharing is not applicable to this article as no datasets were generated or analysed during the current study.
