# Supplementary material for: Frequency and characteristics of errors by artificial intelligence (AI) in reading screening mammography: a systematic review
Source: Breast Cancer Res Treat. 2024 Jun 9;207(1):1–13. doi: 10.1007/s10549-024-07353-3 (PMC11230971; doi:10.1007/s10549-024-07353-3)
Supplement: Supplementary file 1 — Supplementary file1 (DOCX 39 KB) [file 10549_2024_7353_MOESM1_ESM.docx]

Supplementary Information (SI)

Article title: Frequency and Characteristics of Errors by Artificial Intelligence (AI) in Reading Screening Mammography: A Systematic Review

Journal name: Breast Cancer Research and Treatment

Authors: Aileen Zeng^a,b^, Professor Nehmat Houssami^a,b^, Dr Naomi Noguchi^b^ , Dr Brooke Nickel^c,d^ ,Dr M. Luke Marinovich^a,b,^

^a^ The Daffodil Centre, The University of Sydney, a joint venture with Cancer Council New South Wales, Sydney, NSW, Australia

^b^ School of Public Health, Faculty of Medicine and Health, The University of Sydney, Sydney, NSW, Australia

^c^ Wiser Healthcare, Sydney School of Public Health, Faculty of Medicine and Health, The University of Sydney, Sydney, NSW, Australia

^d^ Sydney Health Literacy Lab, Sydney School of Public Health, Faculty of Medicine and Health, University of Sydney, Sydney, NSW, Australia

Corresponding Author: Dr Luke Marinovich

Email: [luke.marinovich@sydney.edu.au](mailto:luke.marinovich@sydney.edu.au)

Online Resource 1: Search Strategy

**MEDLINE**

**Search Date: 23 May 2022**

Ovid MEDLINE(R) ALL <1946 to May 20, 2022>

1 exp Breast Neoplasms/ 326557

2 (breast adj2 (cancer* or tumour* or tumor* or carcinoma* or neoplasm* or disease*)).mp. 435866

3 1 or 2 436011

4 exp Mammography/ 32275

5 mammogram*.mp. 10247

6 4 or 5 34969

7 screen*.mp. or exp mass screening/ 972773

8 exp "Early Detection of Cancer"/ or early detect*.mp. 103976

9 7 or 8 1036365

10 3 and 6 and 9 15228

11 (Artificial intelligence or machine learning or deep learning or neural network*).mp. 179781

12 exp artificial intelligence/ 146182

13 exp Neural Networks, Computer/ 47140

14 11 or 12 or 13 227733

15 10 and 14 413

16 limit 15 to yr="2010 -Current" **345**

**EMBASE**

**Search Date: 23 May 2022**

Embase Classic <1947 to 1973>

Embase <1974 to 2022 May 20>

1 exp breast tumor/ 610584

2 (breast adj2 (cancer* or tumour* or tumor* or carcinoma* or neoplasm* or disease*)).mp. 681365

3 1 or 2 693421

4 exp Mammography/ 63184

5 mammogram*.mp. 14121

6 4 or 5 65219

7 screen*.mp. or exp Mass Screening/ 1562915

8 exp "Early Detection of Cancer"/ or early detect*.mp. 119222

9 7 or 8 1647689

10 3 and 6 and 9 24227

11 (Artificial intelligence or machine learning or deep learning or neural network*).mp. 218924

12 exp artificial intelligence/ 60884

13 exp artificial neural network/ 68638

14 11 or 12 or 13 237183

15 10 and 14 621

16 limit 15 to yr="2010 -Current" **556**

**SCOPUS**

**549 document results**

( TITLE-ABS-KEY ( ( "breast cancer" OR ( breast W/ ( cancer* OR tumour* OR tumor* OR carcinoma* OR neoplasm* OR disease* ) ) ) ) AND TITLE-ABS-KEY ( mammogra* OR "mammography" ) AND TITLE-ABS-KEY ( "screening" OR "early detect*" ) AND TITLE-ABS-KEY ( "artificial intelligence" OR "machine learning" OR "deep learning or neural network*" ) ) AND PUBYEAR > 2009

**Arvix.org**

**Showing 1–50 of 96 results** June 2022 | AI Errors SR Protocol _V6

7

Search v0.5.6 released 2020-02-24 ( arxiv.org”)

Query: order: -announced_date_first; size: 50; include_cross_list: True; terms: AND all=Mammography OR Mammogram* OR "breast cancers" OR "breast neoplasm" OR "breast neoplasms" OR "breast tumor" OR "breast tumors"; AND all="artificial intelligence" OR "machine learning"

Online Resource 2: Eligibility Criteria of Included Studies

The criteria for eligibility for the review are listed below with reference to specific study (i.e. PICO, study design, setting, time frame) and report characteristics (such as years considered, language, publication status).

Inclusion criteria:

- Participants: Enrolled women who participated in screening mammographic examinations from real world clinical settings to independently evaluate AI system(s) for cancer detection accuracy
- Intervention/Index test: Diagnostic Accuracy Studies (Cohort or Cross Sectional) published after 1 January 2010 that report intervention as an AI algorithm for automated interpretation of mammograms, alone or in combination with radiologists to detect breast cancer
- Outcomes: Studies that report any of the following outcomes:

1. False negative – Cancer is not detected by AI System but detected by Human Reader or found at follow-up

2. False positive – incorrectly indicates presence of a suspicious finding (in cases where no cancer is found)

3. Mis-localisation – positive/correct diagnosis of cancer but detected by AI in the wrong location

4. Technical error – failure of AI to interpret the mammogram and output a finding (i.e. computing error, algorithm won’t run, or other technical errors reported)

5. Other errors not captured by this classification

Exclusion criteria:

- Participants:

1. Participants in a non-screening setting (e.g. assessment, diagnosis of symptomatic women)

2. Cancer types other than breast cancer

- Intervention/Index test:

1. Computer aided detection (i.e. not contemporary AI)

2. AI used for imaging modalities other than mammography (i.e. MRI, US or Breast tomosynthesis)

- Mammograms are not utilized in the data set or <95% of images used were completed full field DM screening mammograms

- Studies that have incomplete images for >5% of the dataset (i.e. when only part of the mammogram is reviewed instead of 2 breasts for complete assessment)

3. AI system reported was used only for assisting radiologist’s reading (not as an independent reader)

- Outcomes:

1. Study does not report AI errors in breast cancer detection (false negative, false positive, mis-location, technical error, other)

2. Outcome is prediction of breast cancer risk rather than cancer detection

3. Outcome is detection of specific cancer subtypes

- Study type:

1. Studies that are not published diagnostic accuracy studies (i.e. conference abstracts, reviews, commentaries, case studies)

2. Accuracy studies that do not use external validation (only internal validation is reported, i.e. accuracy is assessed in a subset of the AI training cohort)

- Language: Full-Text Articles not written in English

Online Resource 3: Modified QUADAS-2 Tool

| **Item** | **Response** |
| --- | --- |
| **PARTICIPANT SELECTION - A. RISK OF BIAS** | |
| Was a consecutive or random sample of patients enrolled? | **Yes** - RCTs and cohort studies (prospective or retrospective) with unenriched (consecutive or random) sampling  **Unclear** - If not stated  **No -** other studies |
| Was a case-control design avoided? | **Yes**  **Unclear**  **No** |
| Did the study avoid inappropriate exclusions? | **Yes** – If inappropriate exclusions were avoided  **Unclear** – if not clearly reported  **No** - Exclusion of more than 10% of the samples for any reason, for example retrospective studies with missing data  **No** - Systematic exclusion of types of women / images (e.g. of dense breasts)  **No** - Exclusion based on outcomes (e.g. exclusion of cancer types, exclusion of interval cancers, exclusion/inclusion based on recall decision) |
| Were the women and mammograms included in the study independent of those used to train the AI algorithm? | For test set studies, this translates as has the test set been clearly described as an external (geographically) validation set?  **No** - Any internal validation (e.g. split sample, cross-validation) or temporal validation  **Unclear** - No details stated about the training set and tuning set  **Yes** - External geographical validation (Test set was sample from a different centre; can be in another country or the same country)  For studies in a clinical context:  **Yes** - If the study is located at different centre(s) to those who provided mammograms used to train and tune the AI algorithm  **Unclear** - If not stated  **No -** If there is any overlap |
| Was the image quality of the mammograms not compromised? | **Yes** Standard image pre-processing practices were used in which image formats and resolutions are homogenized for the purpose of training and/or clinical application  **Unclear** – if there is no mention on image quality or information on image metadata  **No**- Image formats and resolutions were altered that could potentially skew and cause variability in readings. (i.e. down or up scaling resolution was used )  **No**- Lack of image metadata (e.g. outdated model of scanner used to acquire imaging data) |
| **Could the selection of patients have introduced bias?** | **RISK: LOW/HIGH/UNCLEAR** |
| **PARTICIPANT SELECTION - B. CONCERNS REGARDING APPLICABILITY** | |
| Is there concern that the included patients do not match the review question? | **High** - If ‘yes’ for any of the following statements  **Unclear** - If no details are provided  **Low** - If ‘no’ for all the following statements   - Not a consecutive or random sample of women attending screening; - Enriched sample / cancer prevalence doesn’t match screening context (>1%); - Mammograms not from full-field digital mammography; - Mammograms not from screening (e.g. diagnostic or symptomatic) or only subset such as recalled cases or false-negatives included - Mammograms were sourced from *open-source repositories* (i.e. concerns of inadvertent duplication of data across repositories, erroneous labelling and incomplete patient demographic) |
| **INDEX TESTS – A. RISK OF BIAS** | |
| If a threshold was used, was it pre- specified? | **Yes -** If using a commercially available AI system which gives a yes/no result, or threshold clearly pre-specified in methods  **Yes -** For systems giving a risk score and study explicitly states the pre-specified threshold  **No -** Using sensitivity / specificity of the reader as benchmark using the same dataset  **No -** Setting the threshold with the validation set without temporal evidence (e.g. published protocol) that threshold was truly pre-specified |
| Could the conduct or interpretation of the index test have introduced bias? | **RISK: LOW /HIGH/UNCLEAR** |
| **INDEX TESTS - B. CONCERNS REGARDING APPLICABILITY** | |
| Is there concern that the index test(s) or comparator, its conduct, or interpretation differ from the review question? | **High** - If ‘yes’ for any of the following   - AI system not yet commercially available, e.g. in house systems; - Study did not use a pre-specified threshold for AI system; - 4 views mammograms was not available to the AI System - human reader had no access to prior mammograms   **Unclear** – If no details are provided  **Low** - If ‘no’ for all of the following   - AI system not yet commercially available, e.g. in house systems; - Study did not use a pre-specified threshold for AI system; - AI system did not have access to 4 views mammograms - human reader had no access to prior mammograms |
| **REFERENCE STANDARD – A. RISK OF BIAS** | |
| Is the reference standard likely to correctly classify the target condition? | **Yes** - If the reference standard is histopathology results from biopsy (cancer present or absent) with at least 2 years follow up to interval cancers AND/OR clinical audits that report radiologists’ decision(s) of correct location of cancer **No** - If the reference standard is histopathology results from biopsy (cancer present or absent) with no follow up or clinical audit on localisation |
| Could the reference standard, its conduct, or its interpretation have introduced bias? | **RISK: LOW /HIGH/UNCLEAR** |
| **REFERENCE STANDARD - B. CONCERNS REGARDING APPLICABILITY** | |
| Is there concern that the target condition as defined by the reference standard does not match the review question? | **High** - If ‘yes’ for any of the following  **Unclear** - If no details are provided  **Low** - If ‘no’ for all of the following   - Length of screening rounds <2 years for follow-up / definition of interval cancers; - Classification not by biopsy/follow-up - Definitive lesion localisation not determined by clinical audit |
| **FLOW AND TIMING – A. RISK OF BIAS** | |
| Did all patients receive a reference standard? | **No** - If there was significant (>10%) loss to follow up for reference standards of interval cancers or subsequent screening results  **No** - If any women who should have received a biopsy or follow-up tests after index test positive results did not receive one or results were unavailable  Unclear- no information was provided  **Yes** - otherwise |
| Did patients receive the same reference standard? | **Yes -** same reference standard was applied to the evaluation dataset & also to the comparator (if relevant)  **No** – Otherwise  **Unclear-** not reported |
| Were all patients included in the analysis? | **Yes -** If there were any exclusions after the point of selecting the cohort, for example intermediate or indeterminate results  **No -** Otherwise |
| Could the patient flow have introduced bias? | **RISK: LOW /HIGH/UNCLEAR** |

Online Resource 4: Algorithm Characteristics of AI systems in included studies

| Study | Machine Learning Technique | Commercial Availability | Data Source for Training & Evaluation | Mammogram Vendors for Training & Evaluation |
| --- | --- | --- | --- | --- |
| AI as Triage | | | | |
| Balta 2020 | Convolutional Neural networks | Yes | Training: NR  Evaluation: Single Centre for Breast Diagnostic Centre; Munich Germany | Training: NR  Evaluation:  Siemens (70%); Hologic (30%) Model: Siemens Mammomat Inspiration & Hologic Selenia Dimensions  4 views (2 views per breast) |
| Lauritzen 2022 | Convolutional Neural networks | Yes | Training: >1 million mammograms from various sites in Europe and USA  Evaluation: Danish (Capital Region) Cancer Screening Program | Training: NR  Evaluation: Siemens (100%); Model: Mammomat Inspiration  4 views (2 views per breast) |
| Lang 2021 | Convolutional Neural networks | Yes | Training: Database of 180,000 normal and 9000 abnormal mammograms  Evaluation: SubCohort from Malmo Breast Tomosynthesis Trial * | Training: 4 different vendors; Hologic; Siemens; General Electric, Waukesha, WI; Philips, Eindhoven, the Netherlands  Evaluation: Siemens (100%) ; Model: Mammomat Inspiration ;  4 views (2 views per breast) |
| Raya-Povedano 2021 | Convolutional Neural networks | Yes | Training: NR  Evaluation: Subcohort from Codoba Tomosynthesis Trial * | Training: NR  Evaluation: Hologic (100%)  Model: Selenia Dimensions  4 views (2 views per breast) |
| AI as Standalone | | | | |
| Larsen 2022 | Convolutional Neural networks | Yes | Training: Mammograms from different screening programs and vendors  Evaluation: Cancer Registry- identified Screening Examinations; 4x Hospitals | Training: Hologic, Selenia Dimensions  Evaluation: Siemens (100%)  Model: Mammomat Inspiration  4 views (2 views per breast) |
| Mayo 2019 | Convolutional Neural networks | No | Training: NR  Evaluation: Tertiary Academic Institution specialising in Cancer HealthCare | Training: NR  Evaluation: Hologic; 4 views (2 views per breast) |
| Schaffter 2020 | Meta-Learning Method (Stacking) | No | Training: Kaiser Permanente Washington Data set linked to Breast Cancer Surveillance Consortium (Prospective Collected Data)  Evaluation: Swedish Breast Cancer Screening Programme (KI) | Training: NR  Evaluation: 4 views (2 views per breast) |

*Screening Trial also examined tomosynthesis but only independent mammography reading results were reviewed; NR= not reported

Online Resource 5: Test of heterogeneity in subgroup (Transpara score) meta-analysis

| Transpara Score | Included Studies | Test of heterogeneity (False Positive Proportion) | Test of heterogeneity (False Negative Proportion) |
| --- | --- | --- | --- |
| 3 | Balta 2020  Lang 2021  Larsen 2022 | Q= 83.50 , df= 2, p<0.01 ; I^2^ =97.5% , τ^2^ =0.01 | Q= 1.53 , df=2, p=0.47 ; I^2^ =0% , τ^2^ =0 |
| 5 | Balta 2020  Lang 2021  Larsen 2022  Lauritzen 2022 | Q= 5153.02, df=3, p<0.01 ; I^2^ =99.9% , τ^2^ =0.10 | Q=22.29 , df=3, p<0.01 ; I^2^ =84.3% , τ^2^ =0.18 |
| 7 | Balta 2020  Lang 2021  Larsen 2022  Raya Povedano 2021 | Q=253.74 , df=3, p<0.01 ; I^2^ =99.3 , τ^2^ =0.03 | Q=3.41 , df=3, p=0.33 ; I^2^ =0% , τ^2^ =0 |
| 9 | Balta 2020  Larsen 2022 | Q= 134.91 , df=1, p<0.01 ; I^2^ =99.3% , τ^2^ =0.04 | Q=8.21 , df=1, p< 0.01; I^2^ =87.8% , τ^2^ =0.27 |

Online Resource 6: Summary of AI test accuracy outcomes

| Study | Screen Detected and/or Interval Cancer | Main Threshold to Detect Cancer | TP | FP | FN | TN | Sensitivity (95% CI) | Specificity (95% CI) | FP Proportion (%) | FN Proportion (%) | AUC |
| --- | --- | --- | --- | --- | --- | --- | --- | --- | --- | --- | --- |
| AI for Triage | | | | | | | | | | | |
| Balta 2020 | Screen detected | Transpara Score >3 | 113 | 13231 | 1 | 4551 | 0.99(0.97,1.00) | 0.26(0.25,0.26) | 73.93(73.28,74.57) | 0.01(0.00,0.04) | NR |
| Balta 2020 | Screen detected | Transpara Score >5 | 109 | 9791 | 5 | 7991 | 0.96(0.92,0.99) | 0.45(0.44,0.46) | 54.71(53.98,55.44) | 0.03(0.01,0.07) | NR |
| Balta 2020 | Screen detected | Transpara Score >7 | 105 | 6135 | 9 | 11647 | 0.92(0.87,0.97) | 0.66(0.65,0.66) | 34.28(33.59,34.98) | 0.05(0.03,0.10) | NR |
| Balta 2020 | Screen detected | Transpara Score >9 | 100 | 2190 | 14 | 15592 | 0.88(0.82,0.94) | 0.88(0.87,0.88) | 12.24(11.77, 12.73) | 0.08 (0.05,0.13) | NR |
| Lang 2021 | Screen detected | Transpara Score > 3 | 66 | 6792 | 2 | 2721 | 0.97(0.93,1.0) | 0.29(0.27,0.30) | 70.89(69.97, 71.79) | 0.02(0.01,0.08) | NR |
| Lang 2021 | Screen detected | Transpara Score > 5 | 61 | 4438 | 7 | 5075 | 0.90(0.82,0.97) | 0.53(0.52,0.54) | 46.32(45.32, 47.32) | 0.07(0.03,0.15) | NR |
| Lang 2021 | Screen detected | Transpara Score >7 | 57 | 2541 | 11 | 6972 | 0.84(0.75,0.93) | 0.73(0.72,0.74) | 26.52(25.65, 27.41) | 0.11(0.06,0.21) | NR |
| Lauritzen 2022 | Screen detected & Interval Cancers | Transpara Score > 5 | 1013 | 41909 | 105 | 71394 | 0.91(0.89,0.92) | 0.63(0.63,0.63) | 36.63(36.35, 36.91) | 0.09(0.08,0.11) | NR |
| Raya- Povedano 2021* | Screen detected & Interval Cancers | Transpara Score > 7 | 100 | 4450 | 13 | 11424 | 0.89(0.81,0.94) | 0.72 (0.71,0.73) | 27.84(27.15,28.54) | 0.08(0.05,0.14) | NR |
| Standalone AI systems | | | | | | | | | | | |
| Larsen 2022 | Screen detected & Interval Cancers | Transpara Score >3 | 933 | 86835 | 24 | 35177 | 0.98(0.97,0.98) | 0.29 (0.29,0.29) | 70.62(70.36,70.87) | 0.02(0.01,0.03) | NR |
| Larsen 2022 | Screen detected & Interval Cancers | Transpara Score >5 | 901 | 61596 | 56 | 60416 | 0.94(0.93,0.96) | 0.50(0.49,0.50) | 50.09(49.81,50.37) | 0.05(0.04,0.06) | NR |
| Larsen 2022 | Screen detected & Interval Cancers | Transpara Score >7 | 862 | 38237 | 95 | 83775 | 0.90(0.88,0.92) | 0.69(0.68,0.69) | 31.09(30.84,31.35) | 0.08(0.06,0.09) | NR |
| Larsen 2022 | Screen detected & Interval Cancers | Transpara Score >9 | 745 | 11638 | 212 | 110374 | 0.78(0.75,0.80) | 0.91(0.90,0.91) | 9.46(9.30,9.63) | 0.17(0.15,0.20) | NR |
| Mayo 2019 | Screen Detected & Interval Cancers | N/A | 3 | 126 | 0 | 116 | 1(1,1) | 0.48(0.42,0.54) | 51.43(45.17, 57.69) | 0 | NR |
| Schaffter 2020 (KI dataset ONLY) | All cancers within 1 year of follow up | Matched to radiologists’  specificity threshold: 77.1 % (single reader); 83.9% (consensus reader) | NR | NR | NR | NR | 0.84 | 0.81 | NR | NR | 0.903 |

*Values extracted from Freeman et al [5] ; NR=not reported; NA= not applicable

Online Resource 7: Summary of test accuracy outcomes of radiologists

| Study | Screen Detected and/or Interval Cancer | Single or Consensus Read | TP | FP | FN | TN | Sensitivity (95% CI) | Specificity (95% CI) | FP Proportion(%) | FN Proportion (%) | AUC |
| --- | --- | --- | --- | --- | --- | --- | --- | --- | --- | --- | --- |
| AI for Triage | | | | | | | | | | | |
| Balta 2020 | Screen Detected | Single Read | 105 | 638 | 9 | 17144 | 0.92(0.87,0.97) | 0.96(0.96,0.97) | 3.56 (3.29,3.84) | 0.05 (0.17,0.83) | NR |
| Balta 2020 | Screen Detected | Consensus Read | 114 | 844 | 0 | 16938 | 1 | 0.95(0.949,0.96) | 4.71 (4.41,5.03) | 0 | NR |
| Lang 2021 | Screen Detected | N/A | NR | NR | NR | NR | NR | NR | NR | NR | NR |
| Lauritzen 2022 | Screen detected & Interval Cancers | Consensus Read | 791 | 2107 | 327 | 111196 | 0.71 (0.68, 0.74) | 0.98 (0.98, 0.98) | 1.84(1.76,1.92) | 0.29(0.25,0.32) | NR |
| Raya- Povedano 2021* | Screen detected & Interval Cancers | Consensus Read* | 76 | 731 | 37 | 15143 | 0.67 (0.58, 0.75) | 0.95(0.95,0.96) | 4.57(4.25,4.90) | 0.23(0.16,0.31) | NR |
| Standalone AI systems | | | | | | | | | | | |
| Larsen  2022 | Screen Detected & interval Cancer | Consensus Read | 752 | 3144 | 205 | 118868 | 0.79(0.76,0.81) | 0.97(0.97,0.98) | 2.56 (2.47,2.64) | 0.17(0.14,0.19) | NR |
| Mayo 2019 | N/A | N/A | N/A | N/A | N/A | N/A | N/A | N/A | N/A | N/A | N/A |
| Schaffter 2020 (KI dataset ONLY) | Screen Detected & interval cancers | Single Read | NR | NR | NR | NR | 0.77 (0.74,0.80) | 0.96 (0.96, 0.96) | NR | NR | NR |
| Schaffter 2020 (KI dataset ONLY) | Screen Detected & Interval Cancers (all cancers withing 1 year of follow up) | Consensus Read | NR | NR | NR | NR | 0.84(0.81,0.87) | 0.98(0.98,0.99) | NR | NR | 0.942 |

*Values extracted from Freeman et al [5] ; NR=not reported; NA= not applicable
